# Quick-Freeze, Deep-Etch Electron Microscopy Reveals the Characteristic Architecture of the Fission Yeast Spore

**DOI:** 10.3390/jof7010007

**Published:** 2020-12-26

**Authors:** Yuhei O. Tahara, Makoto Miyata, Taro Nakamura

**Affiliations:** 1 Department of Biology, Graduate School of Science, Osaka City University, Sumiyoshi-ku, Osaka 558-8585, Japan; tahara@sci.osaka-cu.ac.jp; 2 The OCU Advanced Research Institute for Natural Science and Technology (OCARINA), Osaka City University, Sumiyoshi-ku, Osaka 558-8585, Japan

**Keywords:** *Schizosaccharomyces pombe*, sporulation, cell wall, eisosome, germination

## Abstract

The spore of the fission yeast *Schizosaccharomyces pombe* is a dormant cell that is resistant to a variety of environmental stresses. The *S. pombe* spore is coated by a proteinaceous surface layer, termed the Isp3 layer because it comprises mainly Isp3 protein. Although thin-section electron microscopy and scanning electron microscopy have revealed the fundamental structure of the spore, its architecture remains unclear. Here we visualized *S. pombe* spores by using a quick-freeze replica electron microscopy (QFDE-EM) at nanometer resolution, which revealed novel characteristic structures. QFDE-EM revealed that the Isp3 layer exists as an interwoven fibrillar layer. On the spore cell membrane, many deep invaginations, which are longer than those on the vegetative cell membrane, are aligned in parallel. We also observed that during spore germination, the cell surface changes from a smooth to a dendritic filamentous structure, the latter being characteristic of vegetative cells. These findings provide significant insight into not only the structural composition of the spore, but also the mechanism underlying the stress response of the cell.

## 1. Introduction

The spore of the fission yeast *Schizosaccharomyces pombe* is a quiescent cell that is highly resistant to various stresses, including heat, digestive enzymes, and organic solvents [1,2]. This resistance is associated with various structural features of the spore. Structural characterization of *S. pombe* spore has been performed mainly by thin-section electron microscopy [3,4,5], which has revealed that the spore wall is more extensive than the vegetative cell wall. The spore wall is also thicker than the vegetative cell wall, and its constituents differ substantially. In the vegetative cell wall, α- and β-glucans and galactomannan are the major components [6,7,8]. By contrast, the spore wall comprises chitosan, in addition to mannan and glucans, and is coated by a proteinaceous layer comprising mainly the Isp3 protein. Since spore mutants with defects in the genes responsible for spore wall formation exhibit sensitivity to stress, the spore wall seems to primarily contribute to the stress resistance of spores [9,10,11,12,13,14,15].

Scanning electron microscopy (SEM) has shown that the *S. pombe* spore has many protrusions projecting outwards [16]. In terms of probing spore structure, SEM provides three-dimensional images, but the chemical fixation needed for samples may change the original structure, and the resolution of SEM is lower than that of transmission microscopy. On the other hand, thin-section microscopy does not produce three-dimensional images.

Quick-freeze, deep-etch replica EM (QFDE-EM) has emerged as a useful tool that can be applied to the visualization of many biological phenomena [17,18]. It is an advanced technology that is used to capture a snapshot of a biological specimen in an active state, with a spatial resolution on the order of nanometers and a time resolution of sub-milliseconds. In this method, the specimen is frozen in less than a millisecond by pressing it against a metal block chilled with liquid helium or nitrogen, resulting in much faster fixation than can be achieved by chemical methods [18]. The frozen specimen is exposed by fracturing and etching, and is then shadowed by platinum. The resulting metal replica provides high contrast, allowing clear visualization from a single image without the need for image averaging, unlike cryo-electron microscopy [17,18]. Therefore, QFDE-EM has considerable advantages, especially when used to visualize low-density images, relative to other methods of transmission electron microscopy. 

In this study, we observed *S. pombe* spores by QFDE-EM at nanometer resolution, enabling us to identify some novel structural features: namely an interwoven fibrillar layer on the spore surface and deep invaginations of the spore cell membrane. We also used QFDE-EM to visualize the germination process. 

## 2. Materials and Methods 

### 2.1. Yeast Strains, Media, and Culture Conditions

The study used *S. pombe* strains MKW5 (wild type) and KN3 (*isp3∆*) [15,19]. Complete (YE) medium was used for vegetative culture. Malt extract (ME) and synthetic sporulation (SSA) media were used for sporulation [20,21]. *S. pombe* cells were grown and sporulated at 28 °C.

### 2.2. Isolation of Spores by Density Gradient Centrifugation

The homothallic haploid strains were grown in ME liquid medium for 7 days. The ascal walls were spontaneously dissolved to liberate single spores. The spores were isolated by using linear 25–55% Urografin (Bayer, Leverkusen, Germany) density-gradient centrifugation as described previously [22].

### 2.3. Quick-Freeze Deep-Etch Electron Microscopy

The isolated spores were washed twice with distilled water, and an aliquot was mixed with a slurry including mica flakes and frozen by a CryoPress (Valiant Instruments, St. Louis, MO, USA) cooled by liquid helium. To prepare a replica, the frozen sample was knife-fractured, and subjected to platinum and carbon rotary-shadowing in a JFDV freeze-etching device (JEOL, Akishima, Japan). The platinum replica was removed by floatation on the surface of hydrofluoric acid, cleaned in commercial bleach, rinsed in distilled water, and transferred to a 400-mesh copper grid as previously described [23,24]. Replicas were viewed under a transmission electron microscope (JEM1010, JEOL) at an acceleration voltage of 80 kV. Electron microscopy images were obtained with a FastScan-F214 (T) charge-coupled device (CCD) camera (TVIPS, Gauting, Germany).

## 3. Results

### 3.1. The S. pombe Spore Is Covered by a Fibrillar Structure of Isp3

**Figure 1:**
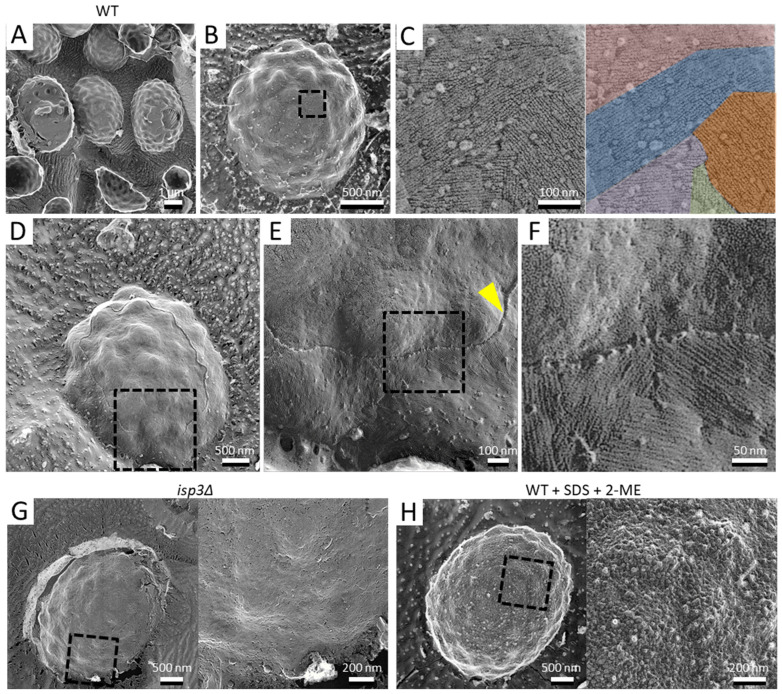
Surface structure of the *S. pombe* spore. (**A**) Field image of wild-type spores. Left spore shows a cytoplasmic cross-section. Center spore shows partial detachment of the surface layer. Right spore shows partial detachment of the spore wall. (**B**) Single spore. (**C**) Left, Magnified image of the boxed region in B. Right, the fibrillar structures are shown in various colors. (**D**) Spore with the outermost layer partly removed by fracture. (**E**) Magnified image of the boxed region in (**D**). Arrowhead indicates a cross-section of the fractured surface layer. (**F**) Magnified image of the boxed region in (**E**). (**G**) Left, *isp3Δ* spore. Right, magnified image of the boxed region. (**H**) Left, wild-type spore pretreated with 1% SDS and 5% β-mercaptoethanol (2-ME). Right, magnified image of the boxed region.

**Figure 2:**
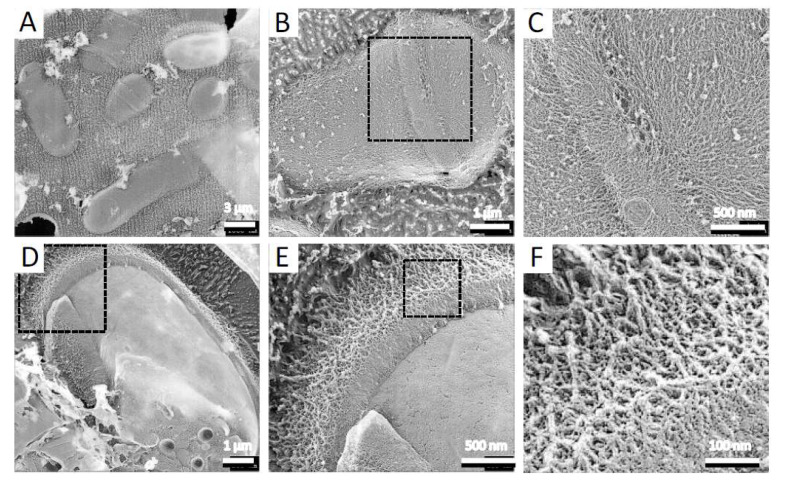
Surface structure of vegetative cell. (**A**) Field image of *S. pombe* wild-type vegetative cells. (**B**) Single vegetative cell. (**C**) Magnified image of the boxed region in B. (**D**) Single fractured cell. (**E**) Magnified image of the boxed region in (**D**). (**F**) Magnified image of the boxed region in (**E**).

To observe the architecture of *S. pombe* ascospores in more detail, we conducted QFDE-EM. Isolated spores (see Materials and Methods) were collected by centrifugation and then mixed with mica flakes. The spores were frozen, fractured, deeply etched, and shadowed with platinum, and then the platinum replicas were recovered and observed (Figure 1). The shape and dimensions of the spores were consistent with those observed in images obtained by SEM [16], and each spore had many protrusions that projected outwards. Whereas dendritic structures covered the surface of vegetative cells (Figure 2), the surface of the spore was essentially smooth (Figure 1A,B). 

Next, we observed the spore surface at a higher resolution, which revealed fibrillar structures with a width of about 7.0 ± 1.9 nm, a length of hundreds of nanometers, and grouped fascicles (Figure 1B,C). The thickness of the layer was about 12.0 ± 3.0 nm (Figure 1D–F). Previous studies have shown that the *S. pombe* spore is covered by a proteinaceous layer, termed the “Isp3 layer”, which comprises mainly Isp3 protein [9,15]. To determine whether the fibrillar structure corresponds to the Isp3 layer, we examined the surface of *isp3∆* spores. QFDE-EM showed that there were no fibrillar structures on the *isp3∆* spore (Figure 1G). 

It is known that Isp3 is firmly associated with the spore wall: extraction of Isp3 from the spore wall requires treatment with both 1% SDS and 5% β-mercaptoethanol, and neither treatment works alone [9,15]. As expected, the fibrillar layer was not observed on spores pretreated with both SDS and β-mercaptoethanol (Figure 1H), similar to *isp3∆* spores. Collectively, these data indicate that the Isp3 layer is present as a fibrillar structure on the spore surface.

### 3.2. Deep Invaginations Are Aligned in Parallel on the Spore Cell Membrane 

**Figure 3:**
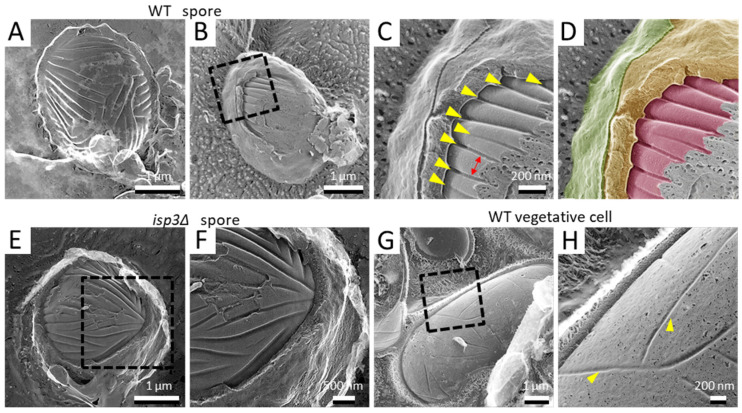
Invagination structure on the membrane surface of spores and vegetative cells. (**A**) Spore with the cell wall detached by fracture. (**B**) Spore with the cell wall and cell membrane partially exposed by fracture. (**C**) Magnified image of the boxed region in B. Yellow arrowheads indicate invaginations of the membrane surface. Arrow indicates the interval of the invaginations. (**D**) Colored image of (**C**). The fibrillar layer is colored green, the spore wall yellow, and the spore cell membrane red. (**E**) *isp3Δ* spore with a fractured spore wall. (**F**) Magnified image of the boxed region in (**E**). (**G**) Vegetative cell surface with fractured cell walls. (**H**) Magnified image of the boxed region in (**G**). Arrowheads indicate invaginations of the membrane surface.

In more deeply fractured samples, we observed spores with the cell wall removed or with the cell membrane exposed (Figure 3A). As described previously, there are many invaginations distributed randomly on the cell membrane of vegetative growing cells ([25]; Figure 3G,H). Using QFDE-EM, many invaginations were also observed on the spore cell membrane. Notably, however, they had a considerably different appearance from those of the vegetative cell membrane: the invaginations of the spore membrane were longer than those of the vegetative cell membrane (Figure 3B–D). In addition, they were aligned in parallel (Figure 3C). The depth and the width were 34.9 ± 4.9 nm and 34.8 ± 6.9 nm, respectively, while the intervals between the invaginations were about 164 ± 34 nm. By contrast, the depth was 32.0 ± 5.3 nm on the vegetative cell membrane. These invaginations were also observed in *isp3∆* spores (Figure 3E,F), indicating that the presence of the Isp3 layer does not affect the formation of these invaginations.

### 3.3. Spore Germination

When appropriate nutrients are supplied, spores germinate to exit dormancy and resume growth. The process of germination involves a marked morphological change from the dormant cell “spore” to the vegetative growing cell. We therefore used QFDE-EM to observe this process. 

**Figure 4:**
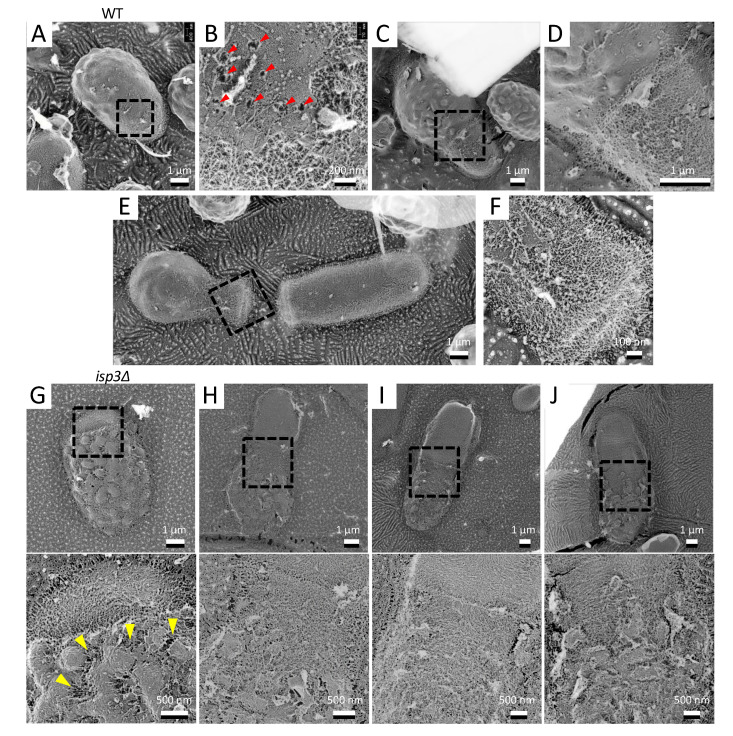
Morphological changes on the spore surface during germination. (**A**) Spore germination of a wild-type cell. (**B**) Magnified image of the boxed region in A. Arrowheads indicate the holes. (**C**) Spore in which germination is proceeding. (**D**) Magnified image in the boxed region in (**C**). (**E**) Image of the first cell division after germination. (**F**) Magnified image of the boxed region in (**E**). (**G**–**J**) Top panels, *isp3Δ* spore germination. Bottom panels, magnified images of the boxed regions above. Arrowheads indicate the collapsed regions of the spore wall.

During germination, *S. pombe* spores develop a polar tube that hatches out of the outer spore wall in a process termed “outgrowth” [25]. QFDE-FM showed that there was no fibrillar layer on the growing tubes, and the inner dendritic structures were exposed (Figure 4A,B). In addition, at the boundary region of the cell surface between the spore and the tube, we frequently observed holes with exposed fibers (Figure 4B). As germination proceeded, the polar tube became coated with the dendritic structure, but the rest of the surface retained the structure normally observed on the spore wall surface (Figure 4C–F).

We also observed the germination process in *isp3∆* spores in which the outermost layer of the spore wall is defective. As germination proceeded, the spore wall of *isp3∆* collapsed faster than that of wild-type spores. Notably, the collapse was clearly observed outside the tip of the protrusion, suggesting that the Isp3 layer plays an important role in maintaining the cell wall during germination. 

## 4. Discussion

### 4.1. The Surface of the S. pombe Spore

In the present study, *S. pombe* spores were visualized at nanometer resolution by using QFDE-EM. First, we found that an interwoven fibrillar layer coats the spore surface, and we showed that this layer corresponds to the Isp3 layer, the outermost proteinaceous layer of the spore wall [9,15]. Interestingly, this fibrillar layer has a very similar appearance to the surface of the conidium, known as the “rodlet layer”, in filamentous fungi [26]. The rodlet layer is a significantly stable protein structure that is resistant to various environmental stresses and comprises a ubiquitously expressed protein named hydrophobin. The primary role of hydrophobins is probably to render the spore surface hydrophobic and water resistant, thereby facilitating spore dispersal in the air [27,28,29]. Notably, however, the Isp3 protein does not show any amino acid sequence similarity to known hydrophobins. Furthermore, the *isp3Δ* mutation does not affect the hydrophobicity of the spore surface (our unpublished data). Thus, although the morphology of the layers formed by Isp3 and hydrophobin is similar, the roles of these layers may be different.

In the budding yeast *Saccharomyces cerevisiae*, the ultrastructure of the spore has been well studied by thin-section microscopy and SEM [30,31,32]. Interestingly, the appearance of the spore surface of *S. cerevisiae* is considerably different from that of *S. pombe*: The *S. cerevisiae* spore has a distinctive ridged or scalloped appearance [30], whereas the *S. pombe* spore has many protrusions. It seems likely that this difference is due to the component of the outermost layer of the spore: *S. cerevisiae* spores are coated by a dityrosine layer that comprises mainly a cross-linked modified di-amino acid, LL-*N*,*N*′-bisformyl dityrosine [33]. In a mutant of the *DIT1* gene responsible for formation of the dityrosine layer, the ridged structure on the spore surface is defective, demonstrating that the ridged structure is composed of the dityrosine polymer itself. In *S. pombe* spores, by contrast, the protrusions still persist on the surface of *isp3∆* spores ([15]; Figure 1G), even though the fibrillar layer is defective, suggesting that the protrusion comprises polysaccharides. 

QFDE-EM also showed that vegetative cells are covered with a dendritic structure. The structure and composition of the cell wall of *S. pombe* vegetative cells are well established. The major structural components are α- and β-glucans, both of which form the framework of the cell wall [7,34,35]. High-resolution SEM of intact cells of *S. pombe* has revealed that the exterior of the vegetative cell wall is primarily composed of α-galactomannoproteins [35], suggesting that the dendritic structure comprises galactomannan. 

We also observed the structural change that takes place on the cell surface during spore germination by using QFDE-EM. As germination proceeded, the spore wall collapsed in the region where the germ tube subsequently forms. Furthermore, we found that a number of holes, through which the inner dendritic structures were exposed, emerged on the germinating spore surface. It is unclear how these holes are formed. The protrusions and the fibrillar layer were retained on the mother cell (i.e., spore) after the first cell division (Figure 4E). Notably, the collapse of the structure occurred faster in *isp3∆* spores than in wild-type spores. Our previous study has described that *isp3∆* spores do not show obvious defects in spore germination (e.g., germination rate and growth speed after germination) [15]. One possibility is that the faster collapse of the spore wall may affect germination under special conditions, including environmental stresses.

### 4.2. Invaginations on the Spore Cell Membrane

Since the yeast cell is covered by the cell wall, QFDE-EM is a very effective method to observe the cell membrane structure in three dimensions. The present study revealed that many long and deep invaginations are aligned in parallel on the spore cell membrane (Figure 3A). This seems to differ essentially from the vegetative cell membrane, where short invaginations are distributed randomly (Figure 3G; [36]). The invaginations on the vegetative cell membrane resemble those seen on the *S. cerevisiae* cell membrane, which are called “eisosomes” [37,38] and act as sites of endocytosis. Therefore, it is possible that the invaginations on the *S. pombe* vegetative cell membrane are similar to the eisosomes of *S. cerevisiae*. However, it seems unlikely that the invaginations on the spore cell membrane function as eisosomes because the spore is a dormant cell. What might be the physiological function of the invaginations on the spore cell membrane? Lee et al., have reported the presence of invaginations on the cell membrane of various fungi, microalgae, and lichens, using QFDE-EM [39]. In most of the organisms that they examined, the invaginations were observed as randomly distributed patterns. Very interestingly, the red algae *Cyanidioshizon merolae* has deep and long invaginations that are aligned in parallel similar to the *S. pombe* spore. *C. merolae* was originally isolated from hot springs and grows under severe conditions, including high temperature (i.e., 50 °C) and acidic conditions (pH 1–2). Given the high resistance of the spore to various stresses, we presume that the deep and long invaginations are involved in survival under conditions of severe stress. 

In summary, by using QFDE-EM, we have identified novel structural features in *S. pombe* spores. In future studies, elucidation of the molecular mechanisms by which these features are formed will unveil their physiological functions.

## References

[1] Egel R. (1977). Selective spore survival during replica-plating of fission yeast. Arch. Microbiol..

[2] Kupiec M., Byers B., Esposito B., Jones E.W., Pringle J.R., Broach J.R. (1997). Meiosis and sporulation in *Saccharomyces cerevisiae*. The molecular and Cellular Biology of the Yeast Saccharomyces.

[3] Yoo B.Y., Calleja G.B., Johnson B.F. (1973). Ultrastructural changes of the fission yeast (*Schizosaccharomyces pombe*) during ascospore formation. Arch. Microbiol..

[4] Tanaka K., Hirata A. (1982). Ascospore development in the fission yeasts *Schizosaccharomyces pombe* and *S. japonicus*. J. Cell Sci..

[5] Hirata A., Shimoda C. (1992). Electron microscopic examination of sporulation-deficient mutants of the fission yeast *Schizosaccharomyces pombe*. Arch. Microbiol..

[6] Osumi M. (1989). Development of methods for observing of bacteria and fungi by electron microscopy. QJM Int. J. Med..

[7] Humbel B.M., Konomi M., Takagi T., Kamasawa N., Ishijima S.A., Osumi M. (2001). In situ localization of β-glucans in the cell wall of *Schizosaccharomyces pombe*. Yeast.

[8] Duran A., Perez P. (2004). Cell wall synthesis. Molecular Biology of Schizosaccharomyces pombe.

[9] Shimoda C. (1983). Existence of a major spore wall protein (23K) in the fission yeast *Schizosaccharomyces pombe*: A possible sporulation-specific protein. Arch. Microbiol..

[10] Martin V., Ribas J.C., Carnero E., Duran A., Sánchez Y. (2000). *bgs2^+^*, a sporulation-specific glucan synthase homologue is required for proper ascospore wall maturation in fission yeast. Mol. Microbiol..

[11] Liu J., Tang X., Wang H., Balasubramanian M. (2000). Bgs2p, a 1,3-β-glucan synthase subunit, is essential for maturation of ascospore wall in *Schizosaccharomyces pombe*. FEBS Lett..

[12] Arellano M., Cartagena-Lirola H., Hajibagheri M.A.N., Duran A., Valdivieso M.-H. (2000). Proper ascospore maturation requires the *chs1^+^* chitin synthase gene in *Schizosaccharomyces pombe*. Mol. Microbiol..

[13] Matsuo Y., Tanaka K., Matsuda H., Kawamukai M. (2005). *cda1^+^*, encoding chitin deacetylase is required for proper spore formation in *Schizosaccharomyces pombe*. FEBS Lett..

[14] Garcia I., Tajadura V., Martín Y.S., Toda T., Sanchez Y. (2006). Synthesis of α-glucans in fission yeast spores is carried out by three α-glucan synthase paralogues, Mok12p, Mok13p and Mok14p. Mol. Microbiol..

[15] Fukunishi K., Miyakubi K., Hatanaka M., Otsuru N., Hirata A., Shimoda C., Nakamura T. (2014). The fission yeast spore is coated by a proteinaceous surface layer comprising mainly Isp3. Mol. Biol. Cell.

[16] Nakamura T., Abe H., Hirata A., Shimoda C. (2004). ADAM family protein Mde10 is essential for development of spore envelopes in the fission yeast *Schizosaccharomyces pombe*. Eukaryot. Cell.

[17] Heuser J.E., Reese T.S., Dennis M.J., Jan Y., Jan L., Evans L. (1979). Synaptic vesicle exocytosis captured by quick freezing and correlated with quantal transmitter release. J. Cell Biol..

[18] Heuser J.E. (2011). The origins and evolution of freeze-etch electron microscopy. Microbiology.

[19] Nakamura-Kubo M., Nakamura T., Hirata A., Shimoda C. (2003). The fission yeast *spo14^+^* gene encoding a functional homologue of budding yeast Sec12 is required for the development of forespore membranes. Mol. Biol. Cell.

[20] Egel R., Egel-Mitani M. (1974). Premeiotic DNA synthesis in fission yeast. Exp. Cell Res..

[21] Moreno S., Klar A., Nurse P. (1991). Molecular genetic analysis of fission yeast *Schizosaccharomyces pombe*. Methods Enzymol..

[22] Nishi K., Shimoda C., Hayashibe M. (1978). Germination and outgrowth of *Schizosaccharomyces pombe* ascospores isolated by Urografin density gradient centrifugation. Can. J. Microbiol..

[23] Miyata M., Petersen J.D. (2004). Spike structure at the interface between gliding *Mycoplasma mobile* cells and glass surfaces visualized by rapid-freeze-and-fracture electron microscopy. J. Bacteriol..

[24] Tulum I., Tahara Y.O., Miyata M. (2019). Peptidoglycan layer and disruption processes in *Bacillus subtilis* cells visualized using quick-freeze, deep-etch electron microscopy. Microbiology.

[25] Hatanaka M., Shimoda C. (2001). The cyclic AMP/PKA signal pathway is required for initiation of spore germination in *Schizosaccharomyces pombe*. Yeast.

[26] Hess W.M., Sassen M.M., Remsen C.C. (1968). Surface characteristics of *Penicillum conidia*. Microbiology.

[27] Wessels J.G. (1996). Hydrophobins: Proteins that change the nature of the fungal surface. Adv. Microbial. Physiol..

[28] Wosten H.A., de Vocht M.L. (2000). Hydrophobins, the fungal coat unravelled. Biochim. Biiophys. Acta.

[29] Linder M.B., Szilvay G.R., Nakari-Setälä T., Penttilä M.E. (2005). Hydrophobins: The protein-amphiphiles of filamentous fungi. FEMS Microbiol. Rev..

[30] Coluccio A., Neiman A.M. (2004). Interspore bridges: A new feature of the *Saccharomyces cerevisiae* spore wall. Microbiology.

[31] Coluccio A., Bogengruber E., Conrad M.N., Dresser M.E., Briza P., Neiman A.M. (2004). Morphogenetic pathway of spore wall assembly in *Saccharomyces cerevisiae*. Eukaryot. Cell.

[32] Neiman A.M. (2011). Sporulation in the budding yeast *Saccharomyces cerevisiae*. Genetics.

[33] Briza P., Kalchhauser H., Pittenauer E., Allmaier G., Breitenbach M. (1996). *N*,*N*’ Bisformyl Dityrosine is an in vivo precursor of the yeast ascospore wall. J. Biol. Inorg. Chem..

[34] Bush D.A., Horisberger M., Horman I., Würsch P. (1974). The wall structure of *Schizosaccharomyces pombe*. Microbiol..

[35] Osumi M. (2012). Visualization of yeast cells by electron microscopy. Microbiology.

[36] Takeo K. (1984). Lack of invaginations of the plasma membrane during budding and cell division of *Saccharomyces cerevisiae* and *Schizosaccharomyces pombe*. FEMS Microbiol. Lett..

[37] Walther T.C., Brickner J.H., Aguilar P.S., Bernales S., Pantoja C., Walter P. (2006). Eisosomes mark static sites of endocytosis. Nat. Cell Biol..

[38] Strádalová V., Stahlschmidt W., Grossmann G., Blažíková M., Rachel R., Tanner W., Malinsky J. (2009). Furrow-like invaginations of the yeast plasma membrane correspond to membrane compartment of Can1. J. Cell Sci..

[39] Lee J.-H., Heuser J.E., Roth R., Goodenough U. (2015). Eisosome ultrastructure and evolution in fungi, microalgae, and lichens. Eukaryot. Cell.

