# Correction: Tahara et al. Quick-Freeze, Deep-Etch Electron Microscopy Reveals the Characteristic Architecture of the Fission Yeast Spore. *J.* *Fungi* 2021, *7*, 7

**DOI:** 10.3390/jof7110930

**Published:** 2021-11-02

**Authors:** Yuhei O. Tahara, Makoto Miyata, Taro Nakamura

**Affiliations:** 1 Department of Biology, Graduate School of Science, Osaka City University, Sumiyoshi-ku, Osaka 558-8585, Japan; tahara@sci.osaka-cu.ac.jp; 2 The OCU Advanced Research Institute for Natural Science and Technology (OCARINA), Osaka City University, Sumiyoshi-ku, Osaka 558-8585, Japan

The authors would like to make the following corrections to this paper [1]:

Figure 1 and Figure 3 should be replaced with the final versions, which were modified according to reviewer’s suggestions.

The correct versions are shown below.

**Figure 1:**
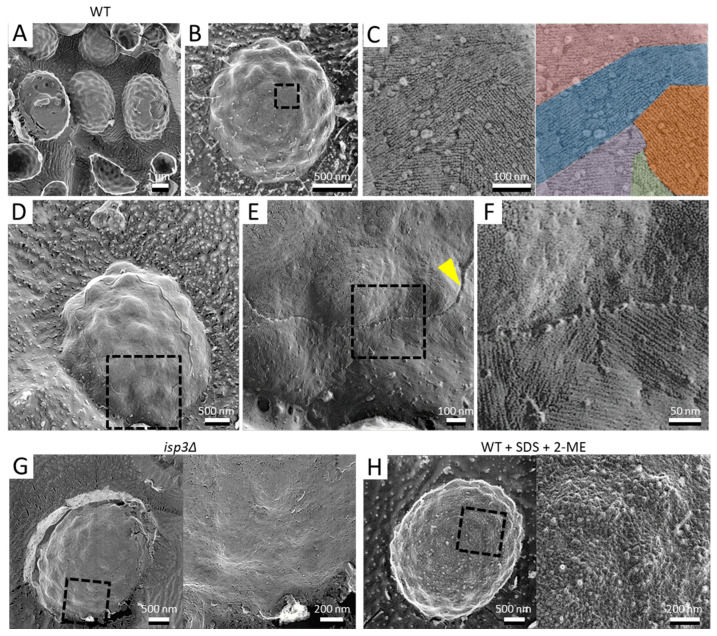
Surface structure of the *S. pombe* spore. (**A**) Field image of wild-type spores. Left spore shows a cytoplasmic cross-section. Center spore shows partial detachment of the surface layer. Right spore shows partial detachment of the spore wall. (**B**) Single spore. (**C**) Left, Magnified image of the boxed region in B. Right, the fibrillar structures are shown in various colors. (**D**) Spore with the outermost layer partly removed by fracture. (**E**) Magnified image of the boxed region in (**D**). Arrowhead indicates a cross-section of the fractured surface layer. (**F**) Magnified image of the boxed region in (**E**). (**G**) Left, *isp3Δ* spore. Right, magnified image of the boxed region. (**H**) Left, wild-type spore pretreated with 1% SDS and 5% β-mercaptoethanol (2-ME). Right, magnified image of the boxed region.

**Figure 3:**
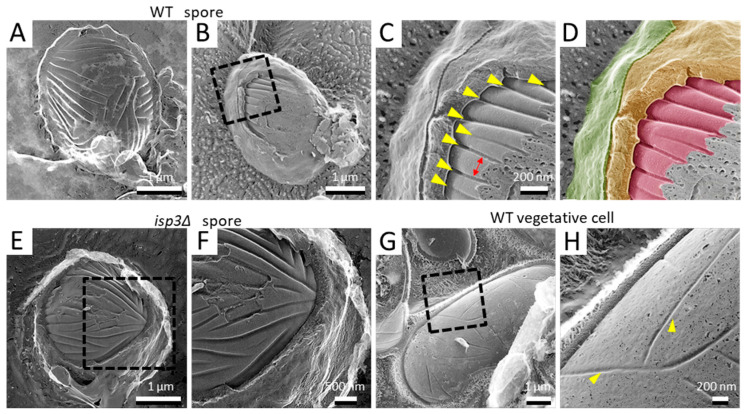
Invagination structure on the membrane surface of spores and vegetative cells. (**A**) Spore with the cell wall detached by fracture. (**B**) Spore with the cell wall and cell membrane partially exposed by fracture. (**C**) Magnified image of the boxed region in B. Yellow arrowheads indicate invaginations of the membrane surface. Arrow indicates the interval of the invaginations. (**D**) Colored image of (**C**). The fibrillar layer is colored green, the spore wall yellow, and the spore cell membrane red. (**E**) *isp3Δ* spore with a fractured spore wall. (**F**) Magnified image of the boxed region in (**E**). (**G**) Vegetative cell surface with fractured cell walls. (**H**) Magnified image of the boxed region in (**G**). Arrowheads indicate invaginations of the membrane surface.
